# Genomic Analysis of 48 *Paenibacillus larvae* Bacteriophages

**DOI:** 10.3390/v10070377

**Published:** 2018-07-19

**Authors:** Casey Stamereilers, Christopher P. Fajardo, Jamison K. Walker, Katterinne N. Mendez, Eduardo Castro-Nallar, Julianne H. Grose, Sandra Hope, Philippos K. Tsourkas

**Affiliations:** 1 School of Life Sciences, University of Nevada Las Vegas, Las Vegas, NV 89154, USA; casey.stamereilers@unlv.edu; 2 Department of Microbiology and Molecular Biology, Brigham Young University, Provo, UT 84602, USA; christopher.fajardo@gmail.com (C.P.F.); jamerkw@byu.edu (J.K.W.); julianne_grose@byu.edu (J.H.G.); sandra_hope@byu.edu (S.H.); 3 Center for Bioinformatics and Integrative Biology, Faculty of the Life Sciences, Universidad Andres Bello, Santiago 8370186, Chile; mendez.katterinne@gmail.com (K.N.M.); Eduardo.castro@unab.cl (E.C.-N.)

**Keywords:** bacteriophages, *Paenibacillus larvae*, American foulbrood, comparative genomics, large terminase, *N*-acetylmuramoyl-l-alanine amidase, major capsid protein

## Abstract

The antibiotic-resistant bacterium *Paenibacillus larvae* is the causative agent of American foulbrood (AFB), currently the most destructive bacterial disease in honeybees. Phages that infect *P. larvae* were isolated as early as the 1950s, but it is only in recent years that *P. larvae* phage genomes have been sequenced and annotated. In this study we analyze the genomes of all 48 currently sequenced *P. larvae* phage genomes and classify them into four clusters and a singleton. The majority of *P. larvae* phage genomes are in the 38–45 kbp range and use the cohesive ends (cos) DNA-packaging strategy, while a minority have genomes in the 50–55 kbp range that use the direct terminal repeat (DTR) DNA-packaging strategy. The DTR phages form a distinct cluster, while the cos phages form three clusters and a singleton. Putative functions were identified for about half of all phage proteins. Structural and assembly proteins are located at the front of the genome and tend to be conserved within clusters, whereas regulatory and replication proteins are located in the middle and rear of the genome and are not conserved, even within clusters. All *P. larvae* phage genomes contain a conserved *N*-acetylmuramoyl-l-alanine amidase that serves as an endolysin.

## 1. Introduction

The gram-positive, spore-forming bacterium *Paenibacillus larvae* is the causative agent of American foulbrood (AFB), the most destructive bacterial disease in honeybees (*Apis mellifera*) and one of the leading causes of their global population decline [1]. Although *P. larvae* exclusively infects honeybee larvae and not adult bees, it is highly virulent and can wipe out an infected hive in a matter of days if untreated [1]. *P. larvae* spores are easily spread by bees and the wind, and thus AFB is highly contagious. Furthermore, *P. larvae* spores are extremely durable, lasting several decades [1]. In recent years several *P. larvae* strains have developed antibiotic resistance, complicating treatment efforts [2,3,4]. Although beekeepers in the United States still have moderate success in treating AFB using the macrolide antibiotic Tylosin Tartrate, in other jurisdictions, such as the European Union, the use of antibiotics in honey is prohibited. If the infection does not clear, incineration of infected hives is the only method for combating *P. larvae* outbreaks.

Given this situation, there has been growing interest in phages that infect and lyse *P. larvae*. The first *P. larvae* phages were identified in the 1950s, with several more isolated in the following decades [5,6,7,8,9,10,11,12,13]. None of these were sequenced at the time, as genome sequencing was still prohibitively expensive and AFB was routinely treated with antibiotics, and thus interest in *P. larvae* phages was scant. However, in the last five years, the number of sequenced *P. larvae* phages has increased significantly, standing at 48 as of this writing. *P. larvae* phages have been isolated and sequenced in Portugal [14], Germany [15], and the United States [16,17,18,19,20,21], with the latter accounting for the vast majority of published genomes. In addition, six studies have been published within the last two years on treating AFB with *P. larvae* phages or *P. larvae* phage endolysins [15,22,23,24,25,26].

The first *P. larvae* phage to have its genome sequenced was phiIBB_Pl23, isolated in Portugal, in 2013 [14], followed by phage HB10c2 in Germany [15], and phages Diva, Lily, Rani, Redbud, Shelly, Sitara and Tripp, isolated in North Carolina [16,17]. In 2015, the genomes of 9 *P. larvae* phages from the University of Nevada, Las Vegas (UNLV) were published [18], followed by the first comparative genomics analysis of *P. larvae* phages [27]. Concurrently, a large number of *P. larvae* phages were isolated at Brigham Young University (BYU) in Utah as part of BYU’s Phage Hunters course over the period 2014–2106, and the genomes of 26 of these were published in 2018 [19,20]. An additional four genomes from UNLV were published in 2018 [21], bringing the total number of published *P. larvae* phage genomes at 48, a number which will continue to grow in the future.

In the present study, we expand upon previous work, give an overview of the genomic landscape of the 48 sequenced *P. larvae* phages, and perform a comparative analysis of their genomes. We group phages into similarity clusters, identify similarities and differences between phages, identify protein functions, and perform comparative analyses on conserved proteins.

## 2. Materials and Methods 

The annotated genomes of phages published by groups other than ours were obtained from NCBI GenBank. For phages isolated by groups at UNLV and BYU, genome assembly was carried out using Geneious 10.2.2 (Biomatters, Auckland, New Zealand) [28] with Medium-Low Sensitivity/Fast and checking for contig circularization. Only phages that produced circularized contigs were annotated and published. 

**Figure 1:**
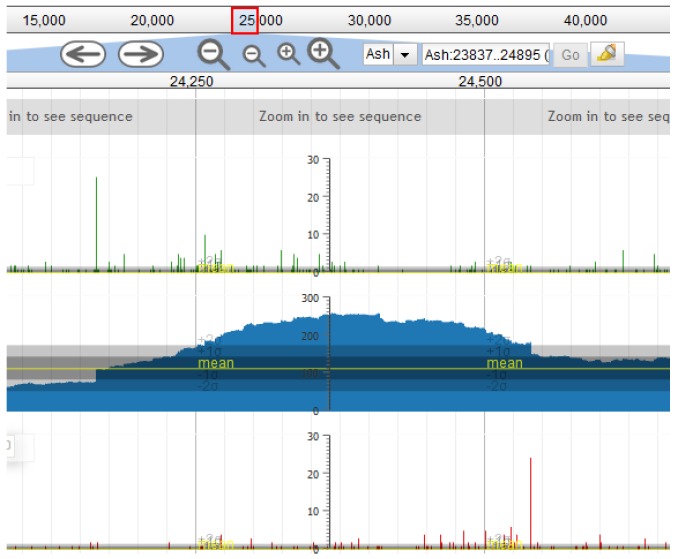
Read coverage map obtained from Pile-up Analysis Using Starts & Ends PAUSE. The direct terminal repeat sequence is located in the sharply delimited region of double coverage depth in clearly visible in the image.

The genome ends and DNA packaging strategy were identified by sequence similarity to previously published *P. larvae* phages and also using the methods described in [29]. First, all BYU and UNLV phages were searched for the known 3′ overhang sequence “CGACTGCCC” near the terminase genes [16,18,19]. If this sequence was found near the terminase genes, the genome was rearranged so that base 1 is the first base after the last “C” of the 3′ overhang sequence. Rearranging the genome in this manner resulted in the start of the small terminase gene being located 50 base pairs (bp) downstream of base 1, consistent with most published *P. larvae* phage genomes. In phages Dragolir, Wanderer and LincolnB, this 3′ overhang sequence was not found, but when these genomes were rearranged as described above, the genome ends were found to contain the sequence “CGACGGCCC”, indicating a point mutation in the 3’ overhang sequence. Phages Ash, Ley, C7Cdelta, Halcyone, Heath, Scottie, and Unity have sequence similarity to phage Tripp [17], which uses the Direct Terminal Repeats (DTR) packaging strategy, and we thus searched these phages for a DTR sequence. The DTR sequence was identified using Pile-up Analysis Using Starts & Ends (PAUSE) (cpt.tamu.edu/computer-resources/pause) and Geneious, looking for a sharply delimited region with double coverage depth, as detailed in [29] and shown in Figure 1. For this method to work, the phage genomes must be sequenced with a PCR-free library preparation method, e.g., Illumina TruSeq (San Diego, CA, USA). 

**Figure 2:**
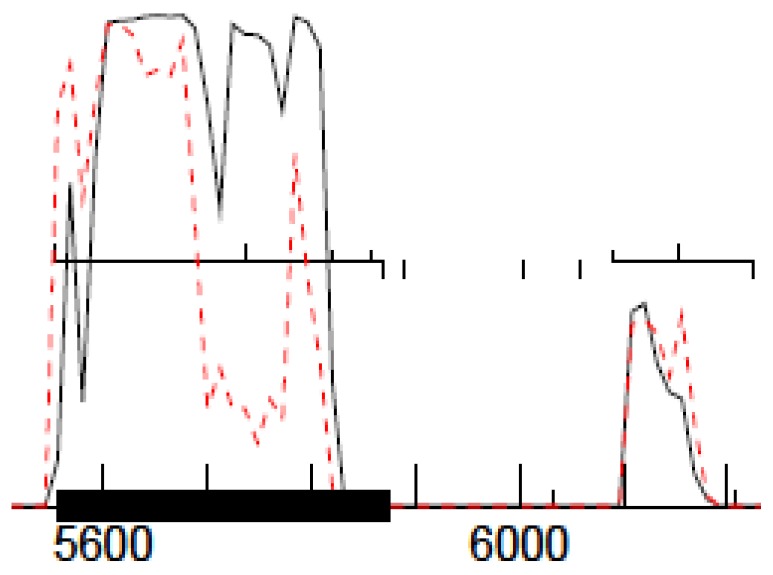
Coding potential map for two open reading frames (ORFs), obtained from GeneMarkS [34]. In the figure, two ORFs are shown, one with strong coding potential (**left**, score: 5) and one with weak coding potential (**right**, score: 1). Upward ticks represent start codons, downward ticks represent stop codons, and horizontal lines between ticks indicate ORFs. The first ORF has four possible start codons, only one of which is located in front of the increase in coding potential (far left).

Annotation of the BYU and UNLV phage genomes was carried out at UNLV using DNA Master (cobamide2.bio.pitt.edu). Open reading frames (ORFs) were assigned a score α that determined whether they were identified as coding based on five criteria: (a) Number of auto-annotation programs that identified the ORF as coding: Glimmer [30] (ccb.jhu.edu/software/glimmer), and the GeneMark family of programs (exon.gatech.edu), specifically GeneMark [31], prokaryotic GeneMark.hmm [32] (host trained with *P. larvae* ATCC 9545), Heuristic GeneMark.hmm [33], GeneMarkS (self-trained) [34], and GeneMark S2 (score can thus range from 0 to 6); (b) existence of coding potential predicted by GeneMarkS as in Figure 2 (score 0–5, based on height and ORF coverage); (c) existence of statistically significant homology matches (BLAST, HMMer) (score 0–5, based on E-value and number of amino acid residues); (d) filling of coding gap and existence of overlaps with other putative genes (score 0–5, based on gap filling and overlap length); and (e) whether the putative gene is part of an operon (start/stop overlaps with the stop/start of an upstream/downstream gene). Criterion (d) takes into account the fact that phage genes seldom overlap (thus overlapping is penalized) and that phage genomes do not have large noncoding gaps (thus gap filling is rewarded). Criterion (e) takes into account the fact that many phages genes are part of an operon, while pseudo-genes are unlikely to be. Putative genes above a cutoff score (typically α = 8) were kept. Genes identified by only one program and genes <200 bp in length were thoroughly investigated as potential false positives. Coding gaps longer than 100 bp were thoroughly scrutinized to check for false negatives.

For each gene, start codons were assigned a score based on (a) the number of auto-annotation programs that chose that start codon (0 to 6); (b) whether a start codon was located in front of the increase in coding potential; (c) the number of amino acid residues in the top homology matches; (d) the length of the resultant gap or overlap, (e) overlap with the stop codon of an upstream gene (indicating an operon); and (f) Shine–Dalgarno score with settings Kibler6 and Karlin Medium in DNA Master. Particular weight was given to criterion (b), with start codons located behind the increase in coding potential significantly penalizart codoned. All of a gene’s start codons were scored in this manner, and the s with the highest score was chosen.

Putative protein function was inferred from searches of the NCBI nonredundant (nr) protein database with BLASTP (blast.ncbi.nlm.nih.gov), searches of the NCBI Conserved Domain Database (CDD) (ncbi.nlm.nih.gov/Structure/cdd) with CD-Search [35], and searches of UniProtKB with HMMer (hmmer.org), using an E-value cutoff of 1 × 10^−3^ for all three. The result with the lowest E-value that was not a “hypothetical protein” was chosen as the putative function. In some instances putative function was assigned by synteny (applicable to structural proteins and tail assembly proteins). Transmembrane domains were identified with TMHMM [36] and tRNAs with Aragorn [37] and tRNAScan [38]. 

Percent average nucleotide identity (ANI) between genomes was obtained by performing a multiple alignment using ClustalW [39] in Geneious, using the IUB cost matrix and default settings. Dot plots were made with Gepard 1.30 (cube.univie.ac.at/gepard) [40]. Gene content analysis was performed by grouping phage proteins into phams with Phamerator using the default (recommended) settings [41] and the results were visualized with SplitsTree [42]. Phage genome maps were generated with Phamerator using the “Align Two Sequences” algorithm of BLASTN and default window and step size, with an E-value cutoff of 1 × 10^−4^ [41]. Proteins phylogenies were constructed by aligning their amino acid sequences using TranslatorX v1.1 (translatorx.co.uk) and Muscle v3.8.31 [43,44]. The best-fit amino acid substitution model was identified using a hierarchical scheme and tested using the Bayesian Information Criterion [45] as implemented in jModelTest2 [46]. Phylogenies were then inferred using a Bayesian inference framework implemented in MrBayes v3.2.6 with two independent runs of 20 million generations each and a sampling frequency of 1000 generations [47]. The first 5000 samples were discarded, and convergence and mixing were evaluated using the average standard deviation of split frequencies and visually inspecting the traces in Tracer v1.6.0 (tree.bio.ed.ac.uk/software/tracer). Trees were midpoint rooted [48] and visualized with FigTree (tree.bio.ed.ac.uk/software/figtree).

## 3. Results

### 3.1. Phage Sources and Geographical Origin 

The phages’ isolation source, geographical origin, isolating institution, year isolated and published, and GenBank accession number are listed in Table 1. For prophages, the geographical origin is that of the isolating institution. All but two of the phages (phiIBB_Pl23 from Portugal, HB10c2 from Germany) were isolated in the United States. Of these, 25 were isolated by the BYU Phage Hunters class, 13 were isolated at UNLV, 7 at North Carolina State University in Raleigh, NC, and 1 at the University of Iowa (phage PBL1c). UNLV phages Harrison, Hayley, Halcyone and Heath were isolated from soil underneath healthy beehives in the Las Vegas area. Phages Vadim, Vegas, and Scottie were isolated from beeswax-containing commercial products (Burt’s Bees) purchased in Las Vegas. Phage Diane was isolated from an American Type Culture Collection (ATCC) culture isolated from an infected bee by White et al. in 1906 [49]. The BYU phages were isolated by students from either bee debris or lysogens, thus the majority, but not all, of these phages are from Utah. Phage PBL1c was isolated in 1984 at the University of Iowa by Dingman et al. [10] and was sequenced in 2018 at BYU. 

### 3.2. P. larvae Phage Systematics

We grouped *P. larvae* phage genomes into clusters using the four approaches in [50]. These are: average nucleotide identity (ANI), gene content similarity, dot plot similarity, and pairwise genome alignment with BLASTN. 

#### 3.2.1. Genome Clustering by ANI

**Figure 3:**
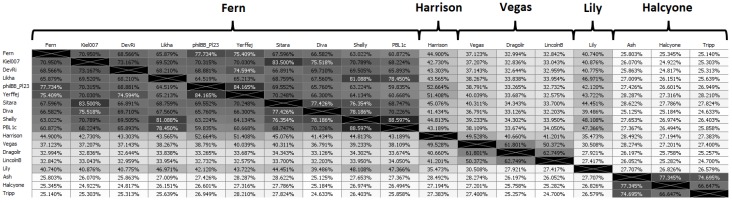
Clusters of *P. larvae* phage genomes determined by average nucleotide identity.

Phages were placed into a cluster if they had greater than 60% ANI with at least one other phage in that cluster [50]. Phages were placed into a subcluster if they had greater than 90% ANI with at least one other phage in that subcluster. Because of the large number of phages, the full ANI distance matrix is large (48 × 48), and we have thus selected a “representative” phage from each subcluster to demonstrate the ANI matrix. The “representative” phage for each subcluster is chosen as the phage with the highest average ANI to all other phages in its subcluster. Singletons (phages that do not belong to a cluster or subcluster) are treated as representatives of their own subcluster. The ANI distance matrix for representative phages is shown in Figure 3, while the full ANI distance matrix is included as Supplementary Figure S1.

There are several instances of phages with ANI > 99.9% to each other (i.e., differ by 40 or less base pairs). The criterion we used to determine whether to publish such phages as separate is whether the differences between their nucleotide sequences produce differences in the corresponding gene products’ amino acid sequences (i.e., phenotypic differences). If that was the case, the phages were published as distinct phages. If not, one of the phages was selected for publication and the other was not published. We found that phages with ANI > 99.975% (i.e., no more than 10 bases different out of 40,000) were phenotypically identical, the sole exceptions to this being phages Fern and Willow (ANI = 99.995%, Figure S1) and Diane, Vadim and Vegas (ANI = 99.989%, Figure S1). On the other hand, if the ANI between two phages was less than 99.975%, this resulted in at least one phenotypic difference.

Rather than an alphanumeric scheme for naming clusters and subclusters, as used previously [27], we named clusters and subclusters after their representative phage, which will allow for easy expansion of clusters should the number of clusters increase to more than 26. By far the largest cluster is the first cluster on the left in Figure 3, named after phage Fern. This cluster contains 30 of the 48 sequenced *P. larvae* phage genomes (Figure S1) and consists of four subclusters (Fern, Rani, DevRi, Xenia) and six singletons. The Fern subcluster is the largest subcluster, containing phages Pagassa, Tadhana, Honeybear, Toothless, Fern, Willow, Lucielle, Saudage, BN12 and Kawika (Figure S1), all of which were isolated at BYU or UNLV. The Rani subcluster contains phages Kiel007, Redbud, Rani, Eltigre and HB10c2. The DevRi subcluster contains phages Arcticfreeze, DevRi, Bloom, Jacopo, Genki, and Gryphonian. This subcluster is extremely tight, as all phages in it have >98% ANI with each other. The Xenia subcluster contains phages Xenia, Shelly, and Leyra. Phages Likha, phiIBB_Pl23, Yerffej, Sitara, Diva and PBL1c are singletons within the Fern cluster, although PBL1c falls just short of the 90% cutoff for inclusion into the Xenia subcluster.

The Harrison cluster consists of phages Harrison and Paisley (Figure S1), while the Vegas cluster consists of a very tight subcluster that contains phages Diane, Hayley, Vadim and Vegas, the singleton phage Dragolir and the LincolnB subcluster consisting of phages LincolnB and Wanderer. The Vegas and LincolnB subclusters are sufficiently different that they would form separate clusters, however as phage Dragolir has >60% ANI with members of both subclusters, it joins the two subclusters together into a single cluster.

Phage Lily is a singleton, with less than 50% ANI with any other *P. larvae* phage genome. The Halcyone cluster contains all the DTR phages, and is comprised of the the Ash subcluster consisting of the BYU phages Ash, C7Cdelta and Ley, the Halcyone subcluster, consisting of UNLV phages Halcyone, Heath, Scottie and Unity, while phage Tripp from North Carolina is a singleton within the cluster. Phage genomes in Halcyone cluster show less than 30% ANI with all other *P. larvae* phage genomes. By comparison, a ClustalW alignment on two randomly generated 40 kbp DNA sequences produces 40% ANI. Phages in the Halcyone cluster are thus very distant from all other *P. larvae* phages.

From these results, it is apparent that geographic origin is not correlated with genome sequence similarity. There are multiple instances of phages from widely different locations having very high ANI (e.g., phages Xenia and Shelly, phages Fern and Willow, phages Diane and Vegas, and phages Redbud and Kiel007 all have >99.5% ANI with each other). Even phages from different continents are found in the same subcluster (e.g., HB10c2 and Kiel007, Rani or Redbud). On the other hand, phages from the same location and even the same isolation source can be very dissimilar from each other (e.g., phages Diva, Lily and Tripp from North Carolina, and phages Halcyone and Harrison from the Las Vegas area).

#### 3.2.2. Phage Clustering by Shared Gene Content Analysis

**Figure 4:**
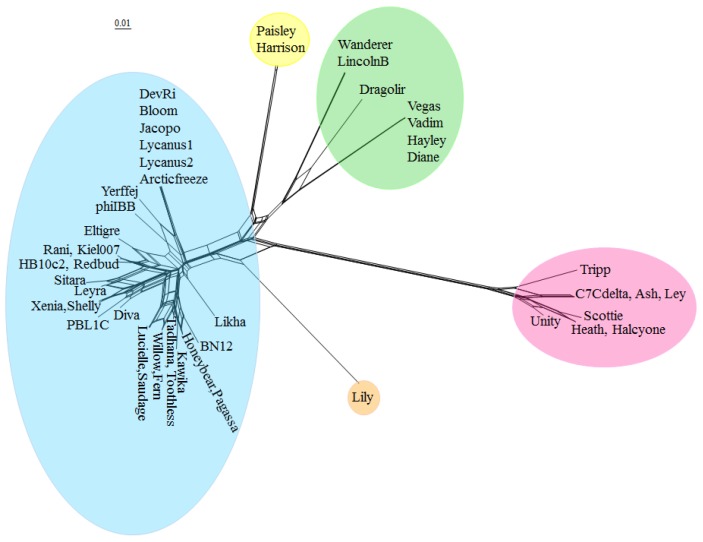
Clustering of *P. larvae* phage genomes based on shared gene content. Genomes were scored for shared gene content using Phamerator and graphed using SplitsTree4. Colored circles indicate the assignment of phages to clusters based on genomic nucleotide content, with blue for the Fern cluster, yellow for the Harrison cluster, orange for the singleton Lily, green for the Vegas cluster, and red for the Halcyone cluster.

Due to the high degree of horizontal gene transfer between phages, construction of phylogenetic trees of complete phage genomes is not appropriate, but an alternative approach is to cluster phage genomes by scoring them based on whether they contain a member of each of the protein phams and visualize the results with SplitsTree [42], shown in Figure 4. Phage genomes were grouped into clusters based on branch length. The clusters generated with this method are identical with the clusters generated by ANI. The complete list of all *P. larvae* phage protein phams and their members is given in Supplementary Table S1.

The low degree of gene content similarity between the Halcyone cluster and the other *P. larvae* phages is reflected by the relatively long length of the Halcyone cluster branch. The high degree of gene content similarity within the Fern and Harrison clusters is reflected by the short branches with those clusters. The large Fern and DevRi subclusters within the Fern cluster are clearly visible. On the other hand, the heterogeneity of the Vegas cluster is reflected by the greater branch length within that cluster.

#### 3.2.3. Phage Clustering by Contiguous Dot Plot Identity

**Figure 5:**
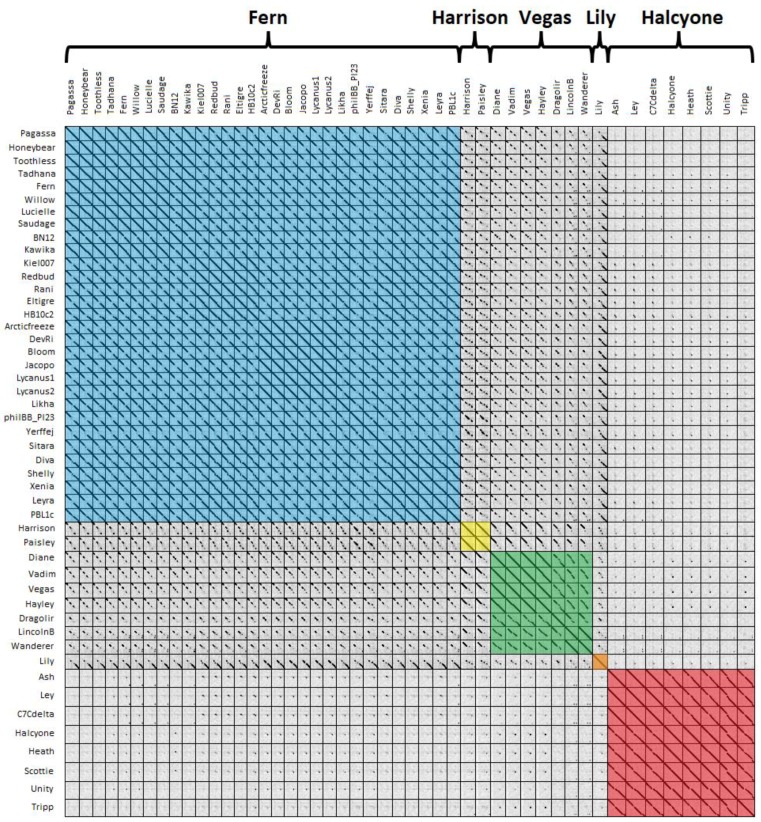
Dot plot of all 48 *P. larvae* phage genomes. Phages are grouped into clusters if they have >50% contiguous dotplot similarity with each other. Phage clusters are highlighted in color, with blue for the Fern cluster, yellow for the Harrison cluster, green for the Vegas cluster, orange for Lily, and red for the Halcyone cluster.

A third way of grouping phages is using dot plots, with phages having >50% contiguous dot plot identity with at least one other phage grouped into the same cluster [50]. A dot plot of all 48 *P. larvae* phage genomes is shown in Figure 5. Clusters are highlighted by color, with blue for the Fern cluster, yellow for the Harrison cluster, green for the Vegas cluster, orange for Lily, and red for the Halcyone cluster.

The clusters generated by dot plot similarity are identical with those generated by ANI and gene content analysis. There is very little genome conservation between the Halcyone cluster and all other *P. larvae* phage genomes, but fairly high genome conservation within that cluster. The Fern cluster phages all have a high degree of genome conservation with each other, particularly at the front and rear of the genome, with the middle of the genome being sometimes divergent. The front end of the genome is conserved across phages in the Fern, Harrison and Vegas clusters, while the rear of the genome is conserved between Lily and the Fern cluster. Phages within the Vegas cluster all have at least 50% contiguous dot plot similarity with each other, despite this cluster being the most heterogeneous.

#### 3.2.4. Phage Clustering with Pairwise Phage Genome Maps

**Figure 6:**
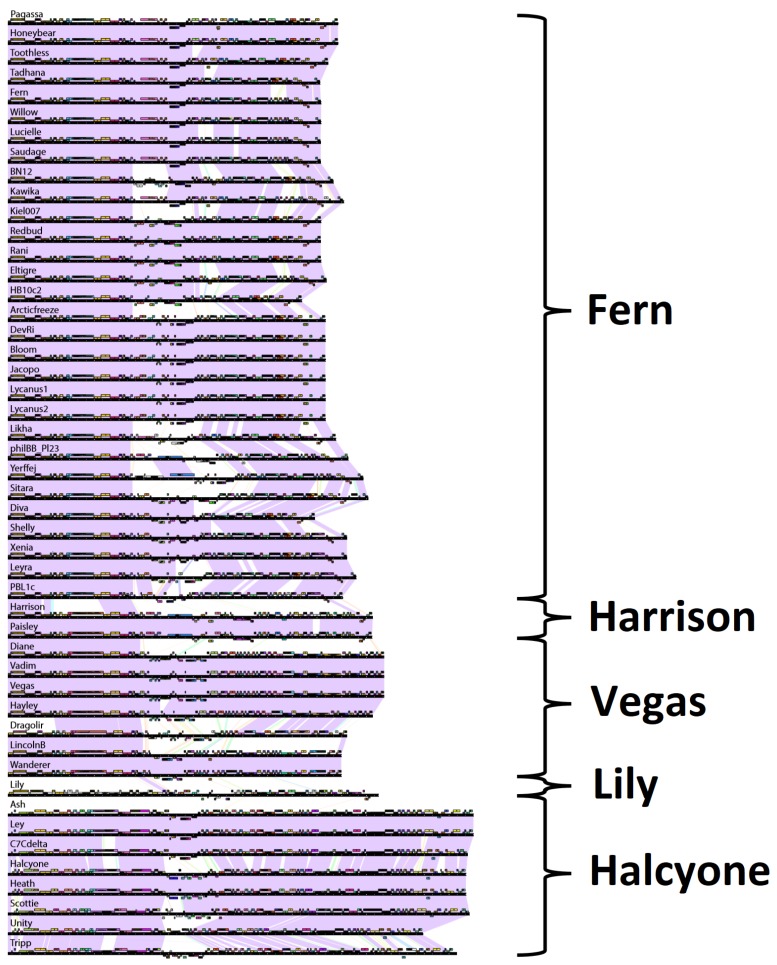
Pairwise genome maps genomes generated with Phamerator. Boxes represent genes, with boxes of the same color indicating genes in the same pham. Genes in a pham of their own (orphams) are uncolored. Shaded areas between genomes indicate regions of high nucleotide sequence similarity between phages as determined by BLASTN, with purple indicating the highest degree of similarity (E-value = 0), and red the lowest (E-value = 1 × 10^−4^).

Another way to represent the relationships between phages and group them into clusters is with pairwise genome map comparisons [50]. Genome maps generated with Phamerator are shown in Figure 6. Purple shading indicates areas of genome sequence similarity between two phage genomes with E-value 0 as determined by the BLASTN “Align Two Sequences” (bl2seq) program. Other colors indicate sequence similarity with higher E-value, with red indicating sequence similarity at the cutoff E-value of 1 × 10^−4^ [48]. We grouped phages into the same cluster if they had greater than 50% BLASTN sequence similarity with each other. Pairwise genome maps are especially useful for showing regions of divergence among similar phages, possible instances of horizontal gene transfer, or gene loss. Regions of high similarity within larger regions of low similarity are indicative of horizontal gene transfer. 

The clusters produced with pairwise genome maps are identical with those of the previous three methods. Within the Fern cluster, approximately the first third of the genome is conserved among all phages in the cluster, while the rear third of the genome is also generally conserved, with the middle region varying between phages in different subclusters. Phage Toothless appears to be missing two genes present in phages Honeybear and Pagassa. There appears to be an instance of horizontal gene transfer between phages Kawika and Kiel and phages philIBB_Pl23 and Yerffej in the middle of their genomes. Phage Fern appears to have a region of divergence towards the rear of the genome with the otherwise highly similar phage Tadhana. There are several instances of this phenomenon within the Fern subcluster. Phage HB10c2 appears to be missing a region present in phages Eltigre and Arcticfreeze. Phages Harrison and Paisley differ by only one gene located towards the rear of the genome. In the Vegas subcluster, phage Hayley is missing two genes found in Diane, Vadim and Vegas but is otherwise highly similar to them. Phage Dragolir is approximately 60% similar with the phages in the Vegas subcluster, and serves as a link between the Vegas subcluster and phages LincolnB and Wanderer, as in Figure S1. Interestingly, there appears to be an instance of horizontal gene transfer between phage Lily and phages Wanderer and LincolnB. The phages within the Halcyone cluster are all fairly similar to one another, with Tripp being the most divergent. Phage Unity appears to be missing a region found in phages Halcyone, Heath, and Scottie. 

### 3.3. P. larvae Phage Genome Characteristics

The key characteristics of the *P. larvae* phage genomes are listed in Table 2. These are genome length, GC content, DNA packaging strategy, number of genes, gene density (genes per 1 kbp), coding fraction, and cluster they belong to. For the UNLV phages Diane, Fern, Harrison, Hayley, Paisley, Vadim, Vegas, Willow and Xenia, the number of genes differs from previous work [18,27] because the genome annotation of these phages was revised with the updated protocol used to annotate the more recently published phages. This also applies to phages phiIBB_Pl23, HB10c2, Diva, Rani, Redbud, Shelly, Sitara, Lily and Tripp [14,15,16,17], which were annotated and published by their respective groups, but were re-annotated by our group using our annotation protocol for the purposes of this paper for consistency.

The length of *P. larvae* phage genomes ranges from a minimum of 35,644 bp (HB10c2) to a maximum of 56,468 (Ash). The majority of genomes (40 of 48) are in the 35–45 kbp range. All phages in the Fern, Harrison, and Vegas clustershave genomes in the 35–45 kbp range and use the 3′ cohesive ends (cos) DNA packaging strategy with 9 bp overhangs. The singleton phage Lily (~45 kbp) uses the 5′ cohesive ends DNA packaging strategy with 12 bp overhangs [16]. The phages in the Halcyone cluster all have genomes in the 50–56 kbp range and use the Direct Terminal Repeat (DTR) DNA packaging strategy, with DTRs either 377 or 378 bp in length. These phages also have a higher GC content than the 3’ and 5’ cohesive ends phages. Coding fraction was calculated by summing all coding gaps, subtracting that from genome length, and dividing the result by genome length. All *P. larvae* phage genomes are ~90–95% coding, with the Vegas subcluster phages being the most highly coding (94.5%), and phage phiIBB_Pl23 the least coding (89.5%).

**Figure 7:**
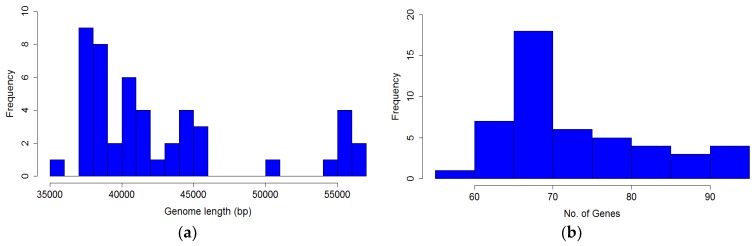
Distribution of (**a**) *P. larvae* phage genome length and (**b**) number of genes in *P. larvae* phage genomes.

The distribution of genome length is shown in Figure 7a, and is clearly bimodal. The distribution of the number of genes in *P. larvae* phage genomes is shown in Figure 7b. The number of genes ranges from 58 (HB10c2) to 91 (Scottie), with a median of 72 genes per genome.

**Figure 8:**
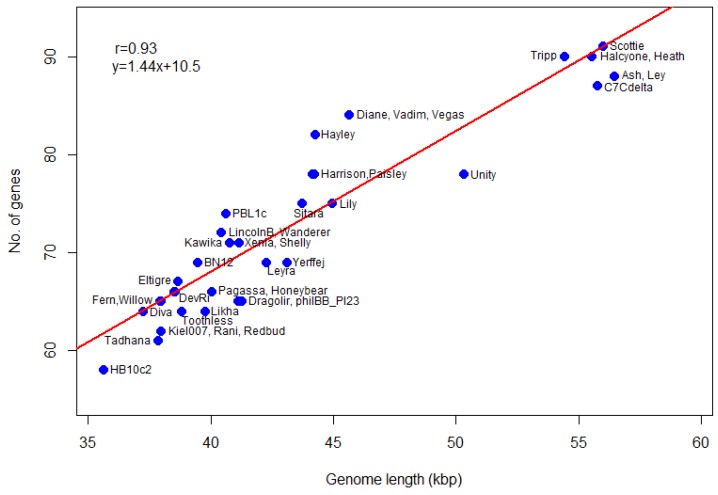
Number of genes in *P. larvae* phage genomes as a function of genome length. The number of genes *y* scales linearly with the genome length in thousands, *x*.

We also calculate gene density (genes per 1000 bp) of *P. larvae* phage genomes. The number of genes as a function of genome length is plotted in Figure 8. The most gene-dense phages are in the Vegas subcluster (Diane, Hayley, Vadim, Vegas), while phage Unity is the least gene dense. The genomes of the Vegas subcluster phages are so gene-dense that they have more genes than the genome of phage Unity, even though the latter is longer. The correlation coefficient between the number of genes and genome length is 0.93, with a *p*-value of 0. A linear regression model fitted to the data predicts a relationship of the form *y = 1.44x + 10.5*, where *y* is the number of genes and *x* the genome length in thousands of base pairs, with *p*-value 0. The number of genes thus scales linearly with genome size. This data can be used in future to check newly annotated genomes for gene overcalling (many false positives) or undercalling (many false negatives).

**Figure 9:**
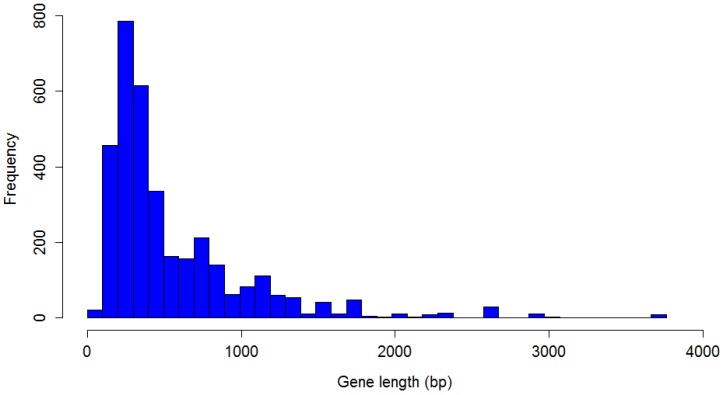
Distribution of *P. larvae* phage gene length.

A total of 3462 genes were identified in the 48 *P. larvae* phage genomes. The distribution of *P. larvae* phage gene length is shown in Figure 9. The distribution is highly non-normal, strongly right-tailed, with significant outliers. The longest identified gene is 3705 bp, the shortest 75 bp, with a median gene length of 375 bp. Nine genes longer than 3000 bp were identified, and 21 shorter than 100 bp. The mode is the 200–300 bp length range (787 genes).

### 3.4. P. larvae Phage Functional Genomics

Statistically significant (E-value < 1 × 10^−3^) homology matches were found for over 90% of *P. larvae* phage proteins with BLAST, HMMer, and CD-Search. A homology match to a protein with putative or known function was found for 1921 (~55%) *P. larvae* phage proteins. Gene products of representative phages from each cluster that have at least one statistically significant homology match to a protein with either putative or known function are shown in Table 3. Cells are colored according to protein function. We classify phage genes into seven functional categories: (1) virion particle (teal); (2) virion assembly (burgundy); (3) host lysis (violet); (4) DNA replication/metabolism (tan); (5) gene regulation, including putative transcription factors (green); (6) host-related functions (yellow); and (7) tRNAs (grey). Gene products whose function cannot be classified into these six categories due to insufficient or conflicting information are left uncolored. Instances of two or more unrelated functions with equally statistically significant matches are marked with a footnote, with the more plausible function listed in the table, and the less plausible function listed in the footnotes at the end of the table. The complete version of Table 3 (all phages) is given in Supplementary Table S2. Gene products with the following functions were identified in all *P. larvae* phage genomes: (1) large terminase; (2) portal protein; (3) major capsid protein; (4 & 5) 2 tail assembly proteins; (6) tail tape measure protein; (7) *N*-acetylmuramoyl-l-alanine amidase; (8) a putative holin; and (9) several tail proteins. In addition, we identified at least one non-identical antirepressor in every genome. Although the overwhelming majority of homology matches were to *P. larvae* proteins, there were also common homology matches to *Geobacillus* and *Brevibacillus* proteins.

#### 3.4.1. Assembly Genes

Proteins involved in phage assembly include the small and large terminase, Clp proteases, and two tail assembly proteins. Genes coding for these functions were identified in most *P. larvae* phage genomes. In some genomes a capsid maturation protease or scaffolding protein was identified. Assembly genes are located at the front of the genome. The small and large terminase are usually the first two genes in the genome, with the large terminase located downstream of the small terminase. A large terminase has been identified in all *P. larvae* phage genomes, but a small terminase was not identified in the Halcyone cluster phages. In several genomes, the small terminase was identified by synteny, when a 300-bp gene was found immediately upstream of the large terminase. All tail assembly proteins were also found by synteny, as none had homology matches to known tail assembly proteins. However, it is known that most *Siphoviridae* have two tail assembly proteins located between the major tail protein and the tail tape measure protein [51,52]. Given that a tail tape measure protein was identified in all *P. larvae* phage genomes, and a major tail protein in almost all genomes, and that in all such instances there are only two genes between the major tail protein and the tape measure protein, we assigned tail assembly function to these two genes. This was further confirmed when we identified a translational frameshift between these genes, as is commonly found in tailed bacteriophages [20,21,22,51,52].

#### 3.4.2. Structural Genes

Structural genes are all located at the front end of the genome in all *P. larvae* phage genomes, comprising the majority of the first 20 genes in the genome. It is likely that genes of unknown function located in the front end of the genome have either structural or assembly function. We identified a portal protein, a major capsid protein, a tail tape measure protein, and several tail proteins in every *P. larvae* phage genome. The tail tape measure protein is encoded by the longest gene in the genome in all of the phages, ranging in length between 2514 bp (Lily) to 3705 bp (Harrison and Vegas clusters). Structural genes are conserved within clusters, however no structural gene is conserved across all clusters. The phams in which the portal protein, major capsid protein, and tape measure protein are distributed, and the pham members are given in Table 4. Each of these three proteins is distributed in four phams. The portal protein and major capsid proteins are identically distributed, with one pham containing the Fern cluster, Harrison cluster, and four of the seven members of the Vegas cluster, one pham containing phages Dragolir, LincolnB and Wanderer, one pham consisting of phage Lily only, and one pham consisting of the DTR phages (Halcyone cluster). For the tape measure protein, one pham consists of the Fern cluster, one pham consists of the Harrison and Vegas clusters, one pham consists of phage Lily only, and one pham consists of the Halcyone cluster.

In a previous study of the Enterobacteriaceae phages, temperate phage nature correlated strongly with major capsid protein (MCP) matches in bacterial chromosomes [53]. A TBLASTN study of the MCP from each of the representative phages supported a temperate nature in that each had a >70% amino acid identity (AAI) match in a bacterial genome (Table 5). 

#### 3.4.3. Host Lysis Genes

All *P. larvae* phage are lytic in vitro, including those isolated from prophages such as Diane, PBL1c, and Xenia. Tailed phages lyse their host by means of a holin/amidase cassette consisting of a hydrophobic holin protein that punctures the host’s inner plasma membrane and a hydrophilic amidase that cleaves the host peptidoglycan wall [54,55,56]. A *N*-acetylmuramoyl-l-alanine amidase has been identified in all *P. larvae* phage genomes. This is the most studied *P. larvae* phage protein, being the subject of at least two studies, and the only *P. larvae* phage protein whose function has been experimentally verified [22,25]. It is the chief gene responsible for lysing *P. larvae,* by cleaving its peptidoglycan cell wall [22,25]. 

A putative holin was identified in all *P. larvae* phage genomes immediately upstream of the *N*-acetylmuramoyl-l-alanine amidase. In most genomes this gene forms an operon with the *N*-acetylmuramoyl-l-alanine amidase. This gene also has strong homology matches to bacteriocin function. The *N*-acetylmuramoyl-l-alanine amidase and putative holin are both distributed in two phams, with the 40 cohesive ends phages in one pham, and the eight DTR phages in the other. All *P. larvae* phage genomes except those in the Halcyone group also encode an additional protein with putative holin function either immediately downstream of the *N*-acetylmuramoyl-l-alanine amidase or two genes downstream. This protein is of the same length as the putative holin located upstream of the *N*-acetylmuramoyl-l-alanine amidase, but also has homology matches to transposase function. Both putative holins have transmembrane domains as predicted by TMHMM and this together with their location in the genome (especially the upstream holin that forms an operon with the *N*-acetylmuramoyl-l-alanine amidase) strongly suggests they may have holin function. 

Phages in the Fern and Vegas clusters also encode a protein near the end of the genome that has homology matches to tranglycosylase function. Transglycosylases, also known as glycosyltransferases, cleave glycosidic bonds in the host glycan, and are thus used by phages for host lysis [57,58]. However the E-value of the homology matches for this protein are only of the order of 1 × 10^−6^, and this gene thus requires further investigation.

#### 3.4.4. DNA Replication and Metabolism Genes

All *P. larvae* phage genomes contain numerous genes with putative functions related to DNA replication and metabolism. These include transposases, integrases, endonucleases, excisionases, methyltransferases, and others. This is by far the largest and most diverse functional category. An integrase has been identified in all but 4 *P. larvae* phage genomes, suggesting that most if not all of these phages possess lysogenic ability. The exceptions are phages Halcyone, Heath, Scottie, and Unity, in whose genomes an integrase was not identified. The majority of *P. larvae* phage genomes also encode at least one transposase, with some phages having multiple different transposases. In addition, many *P. larvae* phages genomes encode an excisionase in the middle of the genome and a HNH endonuclease at the very end of the genome. None of the DNA replication/metabolism genes are found in all genomes or conserved across clusters, and are often not conserved even within clusters.

#### 3.4.5. Regulatory Genes

All *P. larvae* phage genomes contain genes that regulate gene expression, whether in the host or the phage itself. These include XRE (xenobiotic response element), Cro/Cl, AbrB (ambiactive repressor) and ArpU (autolysin regulatory protein) family transcriptional regulators. In addition all phages encode one or more different antirepressors. Many regulatory proteins, in particular the XRE-family transcriptional regulators, contain a helix-turn-helix domain. However, little is known about the function of these proteins in the *P. larvae* phage life cycle. Regulatory genes are the least conserved genes in *P. larvae* phage genomes, with no regulatory gene found in all genomes, or conserved across or within clusters.

#### 3.4.6. Host-Related Genes

*P. larvae* phage genomes encode a variety of host-related proteins, such as toxins, ABC transporters, stress proteins, metallo-hydrolases, toxin–antitoxin systems, and others. Host-related genes are highly heterogeneous with none of these genes widespread among *P. larvae* phage genomes. The sole exception is a toxin–antitoxin system found in all genomes except Lily. The toxin–antitoxin system, while widespread, is not conserved, being distributed into several phams. In some genomes the HicA toxin gene is located in front of the HicB antitoxin gene, while the opposite is true in other genomes.

#### 3.4.7. tRNA Genes

A single tRNA gene was found in the genome of phage Dragolir. This is a tRNA-Pro(tgg) gene coding for a tryptophan tRNA. No tRNA genes were identified in any other *P. larvae* phage genome. 

### 3.5. Comparative Analysis of P. larvae Large Terminase and DNA Packaging Strategy

**Figure 10:**
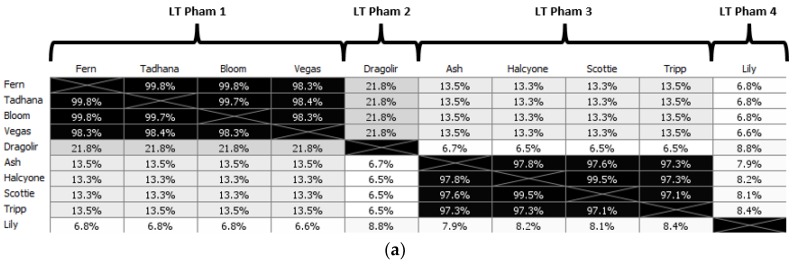

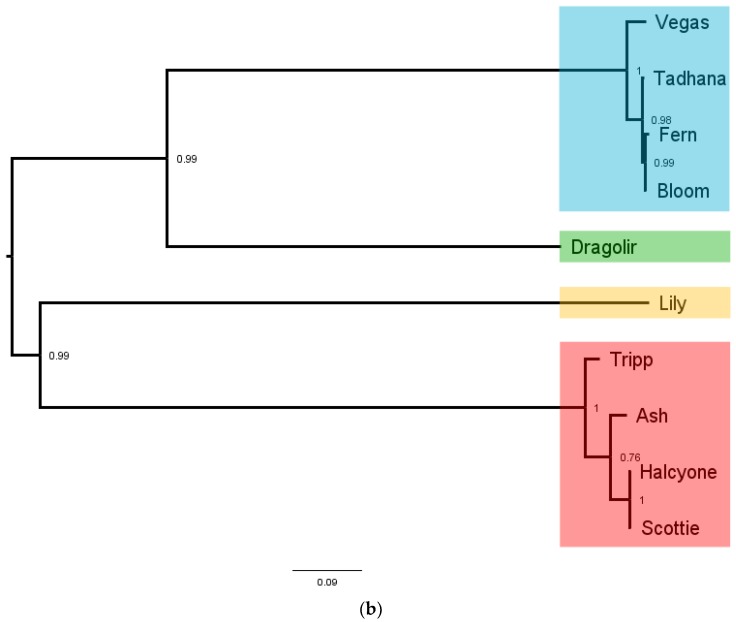
Amino acid sequence identity (AAI) distance matrix of the 10 distinct *P. larvae* phage large terminases (**a**) and corresponding phylogenetic tree (**b**). Large terminases are distributed into four phams, with each pham in the phylogenetic tree highlighted in a different color. LT pham 1 (Fern and Harrison clusters, Diane, Hayley, Vadim, Vegas) is highlighted in blue, LT pham 2 (Dragolir, LincolnB, Wanderer) in green, LT pham 3 (Halcyone cluster) in red, and LT pham 4 (Lily) in orange. Phages in LT pham 1 and LT pham 2 (blue and green, respectively) use the 3′ cohesive ends DNA packaging strategy, while phages in the LT pham 3 (red) use the direct terminal repeats strategy, and phage Lily (orange) uses the 5′ cohesive ends strategy.

As the protein responsible for packaging newly produced phage genomes into empty capsids, the large terminase (LT) is central to the *P. larvae* phage life cycle. A large terminase has been identified in every *P. larvae* phage genome, but there are only 10 distinct large terminases, as many closely related phages have identical large terminases. We constructed a multiple alignment of the 10 distinct *P. larvae* phage large terminases, and the resulting amino acid sequence identity (AAI) distance matrix and phylogenetic tree shown in Figure 10. The full AAI distance matrix of all 48 *P. larvae* phage large terminases is included as Supplementary Figure S2. 

The 10 distinct *P. larvae* phage large terminases are distributed in four phams, in a manner identical to the portal protein (Table 4), with which the large terminase forms an operon. LT pham 1, the largest pham, consists of the large terminases of the phages in the Fern and Harrison clusters, and phages Diane, Vadim, Vegas and Hayley, LT pham 2 consists of the large terminases of phages Dragolir, Wanderer and LincolnB, LT pham 3 consists of the large terminase of phage Lily, and LT pham 4 consists of the large terminases of the Halcyone cluster. 

The large terminases in LT pham 1 all have >98% AAI with each other (Supplementary Figure S2). Of the 30 phages in the Fern cluster, 23 share an identical large terminase, represented in Figure 10 by Fern. The large terminase shared by phages Tadhana, Honeybear, and Toothless differs by one amino acid from the large terminase of Fern but is of the same length (574 aa), implying a silent point mutation. Similarly, the large terminase of phages Bloom, Genki and Gryphonian also differs from that of Fern by a single amino acid (albeit a different one than the large terminase of Tadhana). Phages BN12, Diane, Hayley, Harrison, Paisley, Vadim and Vegas (represented by Vegas in Figure 10) share an identical large terminase that differs by 10 amino acids from those of the Fern cluster. The presence of phage BN12, a Fern cluster phage, in this group is unexpected, as this phage has more than 90% ANI with phages in the Fern cluster and less than 50% ANI with phages in the Harrison and Vegas clusters, yet its large terminase is identical to that of the Harrison and Vegas cluster phages.

LT pham 2 consists of the large terminase shared by phages Dragolir, LincolnB and Wanderer, which is considerably shorter (530 aa) than and highly divergent from the large terminases of LT pham 1. This is despite the fact that phages Dragolir, LincolnB and Wanderer use the same DNA packaging strategy as the phages in LT pham 1 (3′ cohesive ends) and are in the same cluster with the Vegas subcluster phages. It is thus apparent that the large terminase amino acid sequence does not align DNA packaging strategy or cluster assignment based on whole-genome sequence similarity. Phages within the same cluster can have dissimilar large terminases (e.g., Vegas and Dragolir), while phages in different clusters may have very similar large terminases (e.g., Fern and Vegas). Additionally, phages with the same DNA packaging strategy may have highly divergent large terminases (e.g., Fern and Dragolir).

LT pham 3 consists of the large terminases of the Halcyone cluster. This pham consists of four distinct large terminases, which are all of the same length (594 aa) and are overall highly similar to one another (greater than 97% AAI). These large terminases are longer than and have only ~12% AAI with the large terminases of LT pham 1 and LT pham 2. Low AAI is expected, as these phages use the DTR DNA packaging strategy. 

LT pham 4 consists of the large terminase of phage Lily, an outlier that has less than 10% AAI with any other *P. larvae* phage large terminase. This value is lower than even the percent AAI between the large terminase of *P. larvae* phages and the large terminase of phages from different hosts (e.g., *Mycobacterium, Brevibacillus*). This is somewhat unexpected, as phage Lily uses the 5′ cohesive ends DNA packaging strategy, and we would expect it to have a large terminase somewhat similar to that of the 3′ cohesive ends phages.

Large terminase phams are highlighted in different colors in the phylogenetic tree (Figure 10b), with LT pham 1 in blue, LT pham 2 in green, LT pham 3 in ref, and LT pham 4 in yellow. The high similarity of the large terminases of the 3′ cohesive ends phage (with the exception of the Dragolir subcluster) is reflected by the short branch lengths in that section of the tree (blue). Similarly, the high similarity of the DTR phage large terminases is reflected in the very short lengths in their region of the tree (red), while the highly divergent large terminase of Lily has the longest branch length.

### 3.6. Comparative Analysis of P. larvae Major Capsid Protein and P. larvae Phage Morphology

**Figure 11:**
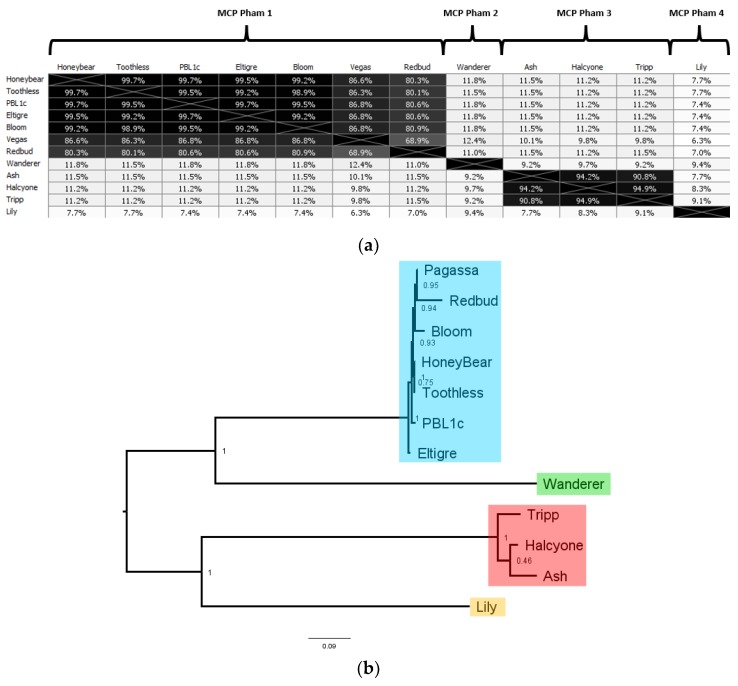
AAI distance matrix of the 10 distinct *P. larvae* phage major capsid proteins (**a**) and corresponding phylogenetic tree (**b**). Major capsid proteins are distributed into four phams, with each pham in the phylogenetic tree highlighted in a different color. MCP pham 1 (Fern and Harrison clusters, Diane, Hayley, Vadim, Vegas) is highlighted in blue, MCP pham 2 (Dragolir, LincolnB, Wanderer) in green, MCP pham 3 (Halcyone cluster) in red, and MCP pham 4 (Lily) in orange.

The major capsid protein is the primary component of the phage capsid and is often used to classify phages, as it is generally conserved between similar phages. A major capsid protein was identified in every *P. larvae* phage genome, with 12 distinct major capsid proteins. The AAI distance matrix and corresponding phylogenetic tree of the 12 distinct *P. larvae* phage major capsid proteins is shown in Figure 11. The full AAI distance matrix of all 48 *P. larvae* phage major capsid proteins is included as Supplementary Figure S3. 

The 12 distinct *P. larvae* phage major capsid proteins are distributed in four phams, in a manner identical to the large terminase and portal protein (Table 4). MCP pham 1, the largest pham, contains the large terminases of the phages in the Fern and Harrison clusters, and phages Diane, Vadim, Vegas and Hayley, MCP pham 2 consists of the major capsid protein of phages Dragolir, Wanderer and LincolnB, MCP pham 3 consists of the major capsid proteins of the phages in the Halcyone cluster, and MCP pham 4 consists of phage Lily. 

In MCP pham 1, Fern cluster phages (except phage Redbud) have one of five distinct major capsid proteins, all of which are highly similar to each other (>99% AAI). This points to the occurrence of point mutations in *P. larvae* phage major capsid proteins. Phages Harrison and Paisley (Harrison cluster), Diane, Hayley, Vadim and Vegas (Vegas cluster) have an identical major capsid protein, which has 87% AAI with those of the Fern cluster, and is thus in the same pham. The high mutual similarity of the major capsid proteins in MCP pham 1 is reflected in the branch lengths in Figure 11b. An outlier in this pham is phage Redbud, whose major capsid has only 81% AAI with the Fern cluster major capsid proteins, even though it has >99% ANI with phages Rani and Kiel007 in the Fern cluster.

MCP pham 2 consists of the major capsid protein of Phages Dragolir, LincolnB and Wanderer. This major capsid protein is very divergent from the major capsid proteins in MCP pham 1 (11% AAI on average), even though these three phages have ~60% ANI with phages in the Vegas cluster. It thus appears that phages in different clusters can have similar major capsid proteins (e.g., Fern and Harrison), but phages in the same cluster can have very dissimilar major capsid proteins (e.g., Vegas and Dragolir). 

MCP pham 3 consists of the major capsid protein of the phages in the Halcyone cluster. This pham consists of 3 distinct major capsid proteins, all with >94% AAI to each other. The major capsid proteins have ~10% AAI with those in the MCP phams 1 and 2. MCP pham 4 consists of the major capsid protein of phage Lily, which is the most divergent of all (>10% AAI with any other MCP). 

**Figure 12:**
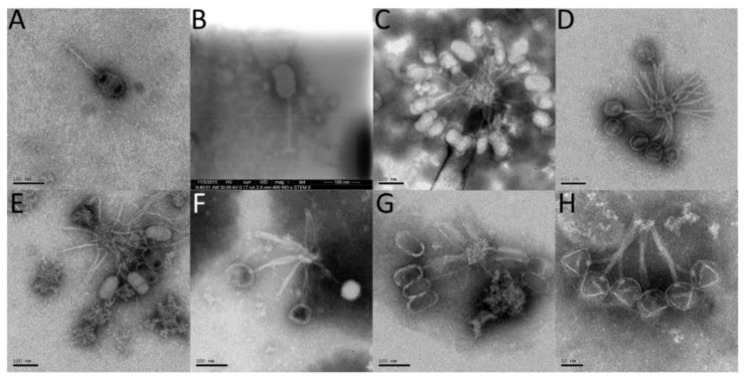
Electron micrographs of phages from the Fern cluster: (**A**) Eltigre; (**B**) Bloom; (**C**) Toothless; (**D**) BN12; (**E**) PBL1c; (**F**) Pagassa from the Vegas cluster: (**G**) Wanderer; and from the Halcyone cluster: (**H**) Ash.

Electron micrographs of several *P. larvae* phages are shown in Figure 12. Additional micrographs of *P. larvae* phages have been published [10,15,20,27]. All known *P. larvae* phages are of the *Siphoviridae* morphotype with long, filamentous, non-contractile tails approximately 150 nm in length. Images in the first six panels of electron micrographs (Figure 12A–F) are phages from the Fern cluster (MCP pham 1), while Figure 12G shows phage Wanderer (Vegas cluster, MCP pham 2) and Figure 12H shows phage Ash (Halcyone cluster, MCP pham 3). Phage PBL1c (Figure 12E) was originally isolated in the 1980s, and the new electron micrograph taken for this publication show an identical virion structure to that in the electron micrographs of this phage taken in 1983 [10]. Of the six phages in MCP pham 1, four (Eltigre, Bloom, Toothless, PBL1c) have prolate capsids approximately 100 nm by 50 nm, while phages BN12 and Pagassa have round capsids approximately 80 nm in diameter. Despite having a different capsid shape, phages BN12 and Eltigre have an identical major capsid protein. The same is true of phages Pagassa and PBL1c. Phage Wanderer has a prolate capsid similar to that of phages Toothless and PBL1c, even though its major capsid protein is in MCP pham 2 and very divergent from those of MCP pham 1. From these results it appears that the amino acid sequence of the major capsid protein does not correlate with capsid shape, as phages with identical major capsid proteins can have different capsid shapes (e.g., Eltigre and BN12), while phages with highly divergent major capsid proteins can have similar capsid shapes (e.g., Wanderer and Toothless). In addition, no differences in the amino acid sequence of the portal proteins were correlated with capsid morphology when examining the Fern cluster. Phage Ash, whose pham is in MCP pham 3, has an icosahedral capsid, wholly different from that of the MCP pham 1 and MCP pham 2 phages. 

### 3.7. Comparative Analysis of P. larvae Phage N-acetylmuramoyl-l-alanine Amidase

**Figure 13:**
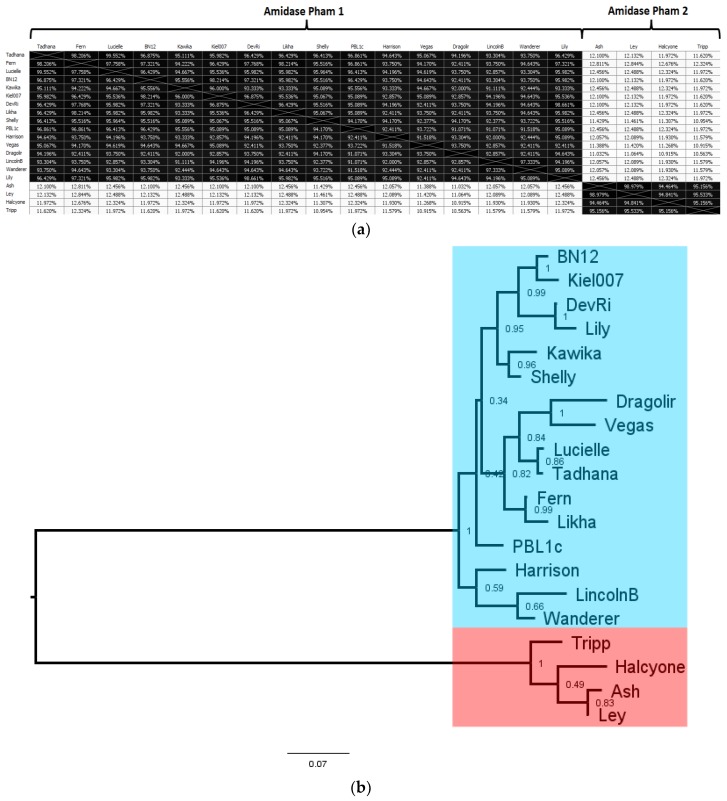
AAI distance matrix (**a**) and phylogenetic tree (**b**) of the 20 distinct *P. larvae* phage *N*-acetylmuramoyl-l-alanine amidases. These are distributed into two phams, one that contains the amidases of all cohesive ends phages (blue), and one that contains the amidases of the Direct Terminal Repeats (DTR) phages (red).

A *N*-acetylmuramoyl-l-alanine amidase was identified in every *P. larvae* phage genome, with 12 distinct *N*-acetylmuramoyl-l-alanine amidases. The AAI distance matrix and corresponding phylogenetic tree of the 12 distinct *N*-acetylmuramoyl-l-alanine amidases is shown in Figure 13. The full AAI distance matrix of all 48 *P. larvae* phage *N*-acetylmuramoyl-l-alanine amidase is included as Supplementary Figure S4. 

The *N*-acetylmuramoyl-l-alanine amidases are distributed in two phams. The two phams are very distinct, with one pham containing the *N*-acetylmuramoyl-l-alanine amidases of the cohesive ends phages (Amidase pham 1) and the other containing the *N*-acetylmuramoyl-l-alanine amidases of the DTR phages (Amidase pham 2). The two phams are highly divergent, with only ~12% AAI between them. On the other hand, the within-pham similarity is quite high: The *N*-acetylmuramoyl-l-alanine amidases of Amidase pham 1 all have greater than 90% AAI with each other, and the *N*-acetylmuramoyl-l-alanine amidases of Amidase pham 2 all have greater than 94% AAI with each other. The divergence between the two phams is reflected in the length of the two main branches of the phylogenetic tree in Figure 13b, while the relative homogeneity of the two phams is reflected by the short length of the branches within each pham. The most divergent of the cohesive ends *N*-acetylmuramoyl-l-alanine amidases are those of phages Harrison, LincolnB, and Wanderer, while the most divergent of the DTR *N*-acetylmuramoyl-l-alanine amidases is that of phage Tripp. Interestingly, while the structural and assembly proteins of phage Lily are very divergent from those of all other *P. larvae* phages, its *N*-acetylmuramoyl-l-alanine amidase is very similar (>90% AAI) with those of the other cohesive ends phages. The *N*-acetylmuramoyl-l-alanine amidase thus appears to be the most conserved gene in the *P. larvae* phage genome. 

The *N*-acetylmuramoyl-l-alanine amidases of the cohesive ends phages (Amidase pham 1) are all in the 223–225 aa length range, thus >90% AAI implies less than 22 amino acids different at most. The *N*-acetylmuramoyl-l-alanine amidases of the DTR phages are all 289 aa long and differ from each other by no more than 15 amino acids at most. Phages within the same subcluster (e.g., the DevRi, Kiel007, Shelly, Vegas, Harrison, and Halcyone subclusters) have identical *N*-acetylmuramoyl-l-alanine amidases with the other members of their subcluster. The sole exception to this is the Fern subcluster, which contains several *N*-acetylmuramoyl-l-alanine amidases that are each slightly different from each other. It thus appears the *N*-acetylmuramoyl-l-alanine amidases generally align with cluster and subcluster assignments based on whole-genome sequence similarity.

## 4. Discussion

In this study, we have analyzed and compared the genomes of all 48 currently sequenced and annotated *P. larvae* phage genomes. This number stood at zero as of 2012, reached 1 by 2014, 18 by 2016, and 48 as of this year. *P. larvae* phages were isolated from sources such as soil underneath hives, beehive products, bees, and beeswax-containing commercial products. A significant number were isolated from *P. larvae* lysogens. Of the 48 sequenced *P. larvae* phages, 46 were isolated in the United States and two in Western Europe. 

A major distinction between *P. larvae* phages can be drawn using the phages’ DNA packaging strategy. Of the 48 *P. larvae* phages, 39 use the 3′ cohesive ends DNA packaging strategy, one uses the 5′ cohesive ends DNA packaging strategy, and eight use the direct terminal repeats (DTR) DNA packaging strategy. Sequence similarity between phages that use different DNA packaging strategies is very low, thus the broadest way to classify *P. larvae* phages is according to the DNA packaging strategy they use.

We place *P. larvae* phage genomes into clusters and subclusters based on four different methods (ANI, gene content, dot plots, BLASTN). Clusters and subclusters are named after the member with the highest average ANI with other cluster members. All four classification methods produce identical results, organizing *P. larvae* phage genomes into four clusters and one singleton. The Fern cluster is the largest cluster, containing 30 of the 48 *P. larvae* phage genomes, while the Harrison cluster contains only two phages and phage Lily is a singleton. It is not known if this discrepancy in cluster size is due to sampling bias or is a reflection of *P. larvae* phage biology. Given that the majority of the 48 *P. larvae* phages were isolated the western United States, it does appear that some *P. larvae* phages are more common than others. On the other hand, geography seems to have no role in the distribution of *P. larvae* phage diversity, as there are multiple of instances of divergent phages isolated from the same narrow geographical area, and phages from widely separated geographical areas showing a very high degree of sequence similarity. 

The overall picture of *P. larvae* phage systematics has both changed somewhat but also remained similar since the last publication in this area [27]. The former cluster A has been split into the Harrison and Vegas clusters, the former still only containing two phages while the size of the latter increased by 3 phages. The former cluster B, now renamed the Fern cluster, still contains the majority of *P. larvae* phages and contains several subclusters and singletons. Previously identified subclusters have grown in size, and a new one (DevRi) has been added, while several phages that were formerly singletons still remain so, and several singletons have been added to this cluster. Phage Lily remains a singleton, while a new cluster, the Halcyone cluster has been added. Phage Lily remains the only *P. larvae* phage that uses the 5′ cohesive ends strategy and is very divergent from all other *P. larvae* phages.

Pairwise genome map comparisons show that within clusters, the front and rear portions of the genome are conserved, while the middle of the genome is often divergent. Pairwise genome map comparisons also reveal several instances of possible horizontal gene transfer between closely related, but also between unrelated phages. Several phages appear to be missing genes present in closely related phages, while some phages appear to be missing large genome regions present in closely related phages. There are also several instances of very closely related phages differing by less than 40 base pairs, with the minimum difference between two phages being only 2 bases (Fern and Willow). Nevertheless, differences of even one base pair are sufficient to result in different amino acid sequences. 

The size of *P. larvae* phage genomes ranges from 35 kbp to 56 kbp, with the majority of genomes in the 38–45 kbp range. A major distinction in genome length exists between the cohesive ends phages and the DTR phages, with the cohesive ends phages having genomes exclusively in the 35–45 kbp range, and the DTR phages having genomes exclusively in the 50–55 kbp range. The DTR phages also have higher GC content. It is not known what role these differences play in the *P. larvae* phage life cycle, as all known *P. larvae* phages are lytic in vitro but appear to be temperate based on close MCP homologs found in bacterial genomes (Table 5). The number of genes in the *P. larvae* phage genome ranges from 58 to 91, with the number of genes scaling linearly with genome size.

Bioinformatics tools identify functions for approximately half of *P. larvae* phage proteins, which is relatively high for bacteriophages. We identified proteins with the following functions in all 48 *P. larvae* phage genomes: (1) large terminase; (2) portal protein; (3) major capsid protein; (4 & 5) two tail assembly proteins; (6) tail tape measure protein; (7) *N*-acetylmuramoyl-l-alanine amidase; (8) a putative holin; and (9) several tail proteins. We classified *P. larvae* phage protein function as (1) virion assembly; (2) structural; (3) lysis; (4) DNA replication/metabolism; (5) regulatory; and (6) host-related. Assembly and structural genes are located at the front of the genome and tend to be conserved within clusters, in common with many tailed phages [59]. Lysis genes are located immediately downstream of structural and assembly genes and also tend to be conserved within clusters. An *N*-acetylmuramoyl-l-alanine amidase gene was identified in all *P. larvae* phage genomes. This gene codes for the *P. larvae* phage endolysin. It is flanked upstream and downstream by two putative holin genes whose products contain a transmembrane domain. However, holins are poorly conserved and difficult to identify bioinformatically as the homology matches of the putative holins are poor. A putative transglycosylase has also been identified in some *P. larvae* phage genomes and could serve as a complementary endolysin. More work is needed to identify the lytic mechanisms of *P. larvae* phages and the genes involved. 

DNA replication/metabolism genes and regulatory genes are located in the middle and rear portions of *P. larvae* phage genomes. Genes of this type are not conserved even within clusters, and their role in the *P. larvae* life cycle is often poorly known. The majority of *P. larvae* phage genomes contain an integrase and one or more transposases, thus it is likely that the majority of *P. larvae* phages have lysogenic ability. This hypothesis is supported by the fact that TBLASTN searches of several phage major capsid proteins show t least a 70% match with bacterial genomes (Table 5). Integrases were identified in all but four phages (Halcyone, Heath, Scottie, and Unity). Halcyone and Heath were isolated from soil underneath healthy hives, making them ideal candidates for treatment of *P. larvae* infections of honeybee hives. All *P. larvae* phage genomes encode host-related genes, with several having potentially significant functions, such as toxins and metallo-hydrolases. 

We performed a comparative analysis of three *P. larvae* phage proteins: the large terminase, the major capsid protein, and the *N*-acetylmuramoyl-l-alanine amidase, each of which was identified in all 48 *P. larvae* phage genomes. The large terminases are distributed into four phams, with one pham containing the majority (34) of the large terminases. Phages with the same DNA packaging strategy tend to have very similar large terminases, often differing by no more than one or two amino acids, while showing very little similarity with the large terminases of phages that employ a different DNA packaging strategy. An exception to this pattern is the large terminase of the 3’ cohesive ends phages Dragolir, LincolnB and Wanderer, which is very divergent from the large terminase of all other *P. larvae* phages that use the 3′ cohesive ends DNA packaging strategy. The major capsid protein is distributed in the same phams as the large terminase. Capsid shape is not correlated with major capsid protein, as phages with the same major capsid protein can have different capsids, and phages with identically-shaped capsids can have divergent major capsid proteins. The *N*-acetylmuramoyl-l-alanine amidases are grouped into two phams, one that contains the *N*-acetylmuramoyl-l-alanine amidases of the *P. larvae* phages that use the cohesive ends DNA packaging strategy and one that contains the *N*-acetylmuramoyl-l-alanine amidases of *P. larvae* phages that use the DTR packaging strategy. Within phams, differences between *N*-acetylmuramoyl-l-alanine amidases tend to be small. It is currently not known why there are two distinct types of *N*-acetylmuramoyl-l-alanine amidase and why they correlate with DNA packaging strategy, and what this means for the *P. larvae* phage the life cycle, as both the DTR and cohesive ends phages are roughly equally competent at lysing *P. larvae.* Of the three proteins comparatively analyzed, the *N*-acetylmuramoyl-l-alanine amidase is the one that most closely aligns with phage cluster grouping based on whole genome sequence similarity, as phages within the same subcluster tended to have very similar *N*-acetylmuramoyl-l-alanine amidases. The *N*-acetylmuramoyl-l-alanine amidase appears to be the most conserved *P. larvae* phage protein, as it is distributed in only two phams, with even the highly divergent phage Lily having a *N*-acetylmuramoyl-l-alanine amidase that is very similar to those of other phages. Point mutations appear to be frequent in *P. larvae* phage genomes, as there are many instances of large terminases, major capsid proteins, and *N*-acetylmuramoyl-l-alanine amidases of different phages differing by only one or two amino acids.

The number of sequenced *P. larvae* phage genomes has grown rapidly in recent years, as they are of great interest in combating AFB. Our comparative genomic study builds on previous work and we expect the field of *P. larvae* phage genomics to grow further in the future. Key areas of further study are: (1) the identification of the function of more *P. larvae* phage proteins, especially in the laboratory as opposed to bioinformatically; (2) precise identification of the mechanisms by which *P. larvae* phages lyse their hosts, including identification of *P. larvae* phage holins and the role of transglycosylase; and (3) the role of phage-encoded beta-lactamases and toxins in *P. larvae* antibiotic resistance and virulence. Other areas of interest are the mechanism by which *P. larvae* phages penetrate their host, the mechanisms by which *P. larvae* phages enter and exit lysogeny, identifying uses of *P. larvae* phage proteins for biotechnology applications, and understanding how *P. larvae* defend against infection from phages.

## Figures and Tables

**Table 1:** Source information for all published *P. larvae* phages. Phages are grouped by the institution responsible for isolating the phage, followed by alphabetic order.

	Isolation Source	Geographical Origin	Institution	Year Isolated/Published	GenBank Accession no.	Ref.
**PBL1c**	Prophage	Iowa City, IA	U. of Iowa	1984/2018	MG727698	[10,19]
**phiIBB_Pl23**	Prophage	Braga, Portugal	Univ. do Minho	2013	KF010834	[14]
**HB10c2**	Dead larva	Celle, Germany	TUB	2015	KP202972	[15]
**Diva**	Honeycomb	North Carolina	NCSU	2015	KP296791	[16]
**Lily**	Honeycomb	North Carolina	NCSU	2015	KP296792	[16]
**Redbud**	Honeycomb	North Carolina	NCSU	2015	KP296794	[16]
**Rani**	Honeycomb	North Carolina	NCSU	2015	KP296793	[16]
**Shelly**	Honeycomb	North Carolina	NCSU	2015	KP296795	[16]
**Sitara**	Honeycomb	North Carolina	NCSU	2015	KP296796	[16]
**Tripp**	Honeycomb	North Carolina	NCSU	2016	KT755656	[17]
**Diane ^1^**	Prophage	Ohio/Las Vegas, NV	UNLV	2013/2015	KT361657	[18]
**Fern ^2^**	Prophage	Germantown, MD	UNLV	2013/2015	KT361649	[18]
**Harrison**	Soil	Las Vegas, NV	UNLV	2013/2015	KT361651	[18]
**Hayley**	Soil	Las Vegas, NV	UNLV	2013/2015	KT361655	[18]
**Paisley**	Soil	Pennsylvania	UNLV	2013/2015	KT361653	[18]
**Vadim ^3^**	Lip balm	Las Vegas, NV	UNLV	2013/2015	KT361656	[18]
**Vegas ^3^**	Lip balm	Las Vegas, NV	UNLV	2013/2015	KT361654	[18]
**Willow**	Soil	Bremerton, WA	UNLV	2013/2015	KT361650	[18]
**Xenia**	Infected larva	Germantown, MD	UNLV	2013/2015	KT361652	[18]
**Halcyone**	Soil	Las Vegas, NV	UNLV	2013/2018	MH460827	[21]
**Heath**	Soil	Las Vegas, NV	UNLV	2013/2018	MH460826	[21]
**Scottie ^3^**	Hand cream	Las Vegas, NV	UNLV	2013/2018	MH460825	[21]
**Unity**	Beehive	Las Vegas, NV	UNLV	2014/2018	MH460824	[21]
**Arcticfreeze**	Bee sample	Idaho	BYU	2016/2018	MH431932	[20]
**Ash**	Prophage	Provo, UT	BYU	2014/2018	MH454076	[20]
**Bloom**	Bee debris	Spanish Bottom, UT	BYU	2015/2018	MH454077	[20]
**BN12**	Bee debris	Cedar City, UT	BYU	2014/2018	MG727695	[19]
**C7Cdelta**	Bee sample	Cedar City, UT	BYU	2014/2018	MH431938	[20]
**DevRi**	Bee debris	Spanish Fork, UT	BYU	2015/2018	MH431933	[20]
**Dragolir**	Bee debris	Wisconsin	BYU	2014/2018	MG727697	[19]
**Eltigre**	Bee debris	South Jordan, UT	BYU	2014/2018	MH454078	[20]
**Honeybear**	Feral bees	Farmington, UT	BYU	2014/2018	MH431935	[20]
**Genki**	Bee debris	Orem, UT	BYU	2014/2018	MH454082	[20]
**Gryphonian**	Bee debris	Orem, UT	BYU	2014/2018	MH431934	[20]
**Jacopo**	Infected hive	Portland, OR	BYU	2016/2018	MH454079	[20]
**Kawika**	Dead bees	Provo, UT	BYU	2015/2018	MH431936	[20]
**Kiel007**	Bee debris	Salt Lake City, UT	BYU	2014/2018	MG727696	[19]
**Ley**	Prophage	Provo, UT	BYU	2014/2018	MH454080	[20]
**Leyra**	Bee debris	Idaho	BYU	2015/2018	MG727701	[19]
**Likha**	Honeycomb	American Fork, UT	BYU	2016/2018	MG727702	[19]
**LincolnB**	Bee debris	Brigham City, UT	BYU	2014/2018	MH454081	[20]
**Lucielle**	Dead bee	Idaho	BYU	2015/2018	MH431937	[20]
**Pagassa**	Prophage	Provo, UT	BYU	2014/2018	MG727699	[19]
**Saudage**	Bee debris	Lehi, UT	BYU	2014/2018	MH454083	[20]
**Tadhana**	Prophage	Provo, UT	BYU	2014/2018	MG727700	[19]
**Toothless**	Bee debris	West Jordan, UT	BYU	2014/2018	MH454084	[20]
**Wanderer**	Bee debris	Wisconsin	BYU	2014/2018	MH431930	[20]
**Yerffej**	Bee debris	Wisconsin	BYU	2014/2018	MH431931	[20]

^1^ Lysogenic phage from ATCC culture 25747 isolated in Ohio by White from an infected bee. ^2^ Lysogenic phage from *P. larvae* wild strain 2231 isolated from an infected larva scale. ^3^ Isolated from commercial products purchased in LasVegas, NV.

**Table 2:** Genomic characteristics of *P. larvae* phage genomes. Phages are grouped by cluster, with the largest (Fern) cluster first, as in Figure S1. Bold indicates the maximum or minimum value of a genomic characteristic. For phages that use the DTR DNA packaging strategy, the length of the direct terminal repeats is shown in parentheses.

	Genome Length (bp)	DNA Packaging Strategy	GC Content (%)	No. of Genes	Genes Per 1000 bp	Coding Fraction (%)	Cluster
**Pagassa**	40,035	3′ cos	42.0	66	1.65	91.2	Fern
**Honeybear**	40,054	3′ cos	41.9	66	1.65	91.2	Fern
**Toothless**	38,832	3′ cos	42.0	64	1.65	91.0	Fern
**Tadhana**	37,880	3′ cos	42.1	61	1.61	91.2	Fern
**Fern**	37,995	3′ cos	41.9	65 ^1^	1.71	91.5	Fern
**Willow**	37,994	3′ cos	41.9	65 ^1^	1.71	91.5	Fern
**Lucielle**	37,947	3′ cos	41.8	65	1.71	91.3	Fern
**Saudage**	37,962	3′ cos	41.9	65	1.71	91.6	Fern
**BN12**	39,485	3′ cos	42.6	69	1.75	92.4	Fern
**Kawika**	40,769	3′ cos	41.6	71	1.74	89.8	Fern
**Kiel007**	37,985	3′ cos	41.8	62	1.63	91.7	Fern
**Redbud**	37,971	3′ cos	41.8	62 ^2^	1.63	91.4	Fern
**Rani**	37,990	3′ cos	41.8	62 ^2^	1.63	91.8	Fern
**Eltigre**	38,675	3′ cos	41.4	67	1.73	92.1	Fern
**HB10c2**	**35,644**	3′ cos	41.8	**58** ^2^	1.63	91.2	Fern
**Arcticfreeze**	38,518	3′ cos	41.5	66	1.71	90.6	Fern
**DevRi**	38,520	3′ cos	41.5	66	1.71	90.6	Fern
**Bloom**	38,519	3′ cos	41.5	66	1.71	90.6	Fern
**Jacopo**	38,526	3′ cos	41.6	66	1.71	90.6	Fern
**Genki**	38,540	3′ cos	40.5	66	1.71	90.6	Fern
**Gryphonian**	38,541	3′ cos	40.5	66	1.71	90.6	Fern
**Likha**	39,778	3′ cos	41.3	64	1.61	92.2	Fern
**phiIBB_Pl23**	41,294	3′ cos	48.1	65 ^2^	1.57	**89.5**	Fern
**Yerffej**	43,126	3′ cos	40.6	69	1.60	90.4	Fern
**Sitara**	43,724	3′ cos	41.6	75 ^2^	1.72	89.6	Fern
**Diva**	37,246	3′ cos	42.1	64 ^2^	1.72	89.6	Fern
**Shelly**	41,152	3′ cos	41.5	71 ^2^	1.73	89.7	Fern
**Xenia**	41,149	3′ cos	41.5	71 ^1^	1.73	89.9	Fern
**Leyra**	42,276	3′ cos	41.4	69	1.63	90.9	Fern
**PBL1c**	40,611	3′ cos	41.2	74	1.82	90.5	Fern
**Harrison**	44,247	3′ cos	40.2	78 ^1^	1.76	91.6	Harrison
**Paisley**	44,172	3′ cos	**40.0**	78 ^1^	1.77	91.4	Harrison
**Diane**	45,653	3′ cos	43.7	84 ^1^	1.84	**94.5**	Vegas
**Vadim**	45,653	3′ cos	43.7	84 ^1^	1.84	**94.5**	Vegas
**Vegas**	45,653	3′ cos	43.7	84 ^1^	1.84	**94.5**	Vegas
**Hayley**	44,256	3′ cos	43.5	82 ^1^	**1.85**	94.2	Vegas
**Dragolir**	41,131	3′ cos	44.0	65	1.58	92.3	Vegas
**LincolnB**	40,437	3′ cos	42.3	72	1.78	93.2	Vegas
**Wanderer**	40,448	3′ cos	42.4	72	1.78	93.2	Vegas
**Lily**	44,952	5′ cos	42.7	75	1.67	90.0	Lily
**Ash**	**56,468**	DTR (377)	48.0	88	1.56	90.9	Halcyone
**Ley**	56,465	DTR (377)	48.0	88	1.56	90.9	Halcyone
**C7Cdelta**	55,774	DTR (377)	48.0	87	1.56	90.9	Halcyone
**Halcyone**	55,560	DTR (377)	48.6	90	1.62	91.8	Halcyone
**Heath**	55,560	DTR (377)	48.6	90	1.62	91.8	Halcyone
**Scottie**	55,990	DTR (377)	48.6	**91**	1.63	91.2	Halcyone
**Unity**	50,316	DTR (378)	**49.1**	78	**1.55**	92.5	Halcyone
**Tripp**	54,439	DTR (378)	48.3	90^2^	1.65	89.6	Halcyone

^1^ Number of genes differs from [18,27] because phages were re-annotated using new criteria for gene calling. ^2^ Number of genes differs from [14,15,16,17] because phages were re-annotated by our group for consistency.

**Table 3:** Gene products of representative *P. larvae* phage genomes with statistically significant homology matches to proteins with known or putative function. Genes are highlighted according to function, with assembly genes in burgundy, structural genes in teal, lysis genes in purple, DNA replication/metabolism genes in tan, regulatory genes in green, host function genes in yellow, and tRNAs in grey. Gene products whose function cannot be classified into these seven categories due to insufficient or conflicting information are left uncolored.

	Fern	Harrison	Vegas	Dragolir	Lily	Halcyone
small terminase	gp1	gp1	gp1	gp1	gp1	gp5
large terminase	gp2	gp2	gp2	gp2	gp2	gp6
portal protein	gp3	gp3	gp3	gp3	gp4	gp7
Clp protease ClpP	gp4	gp4	gp4		gp5	
capsid maturation protease				gp4		
minor capsid protein						gp8
Clp protease ClpB						gp9
major capsid protein	gp5	gp5	gp5	gp5	gp7	gp10
head-tail connector complex	gp7	gp7	gp7	gp6		
head-tail connector complex	gp8	gp8	gp8	gp8		
tail protein	gp10				gp10	
tail sheath protein					gp13	
tail tube protein					gp14	
major tail protein	gp11	gp11	gp11	gp11		
tail assembly protein	gp12	gp12	gp12	gp12	gp15	gp16
tail assembly protein	gp13	gp13	gp13	gp13	gp16	gp17
tail tape measure protein	gp14	gp14	gp14	gp14	gp17	gp18
tail protein	gp15	gp15	gp15	gp15	gp18	gp19
late control protein					gp19	
baseplate assembly protein					gp20	
head-tail connector protein					gp21	
baseplate wedge					gp22	
baseplate protein					gp23	
tail protein	gp16	gp16	gp16	gp16	gp24	gp20
tail protein	gp17	gp17	gp17	gp17	gp25	
putative holin bhlA	gp20 ^1^	gp20 ^1^	gp20^1^	gp20 ^1^	gp28 ^1^	gp22
*N*-acetylmuramoyl-l-alanine amidase	gp21	gp21	gp21	gp21	gp29	gp23
putative holin	gp22 ^2^	gp23 ^2^	gp23 ^2^	gp22 ^2^	gp30 ^2^	
ABC-like transporter protein		gp24				
transposase			gp30			
putative toxin-like protein	gp24					
transcriptional regulator MarR	gp28					
toxin		gp27		gp24		
XRE family transcriptional regulator				gp25		
transcriptional regulator		gp31				
ankyrin-repeat containing protein			gp31 ^3^			
Arc-like DNA binding protein			gp32			
putative holin-like toxin			gp33			
Cro/C1 family transcriptional regulator				gp33		gp26
site-specific recombinase						gp27
stress protein			gp35			
integrase	gp29		gp37	gp37		
metallo-endopeptidase ImmA/Irre			gp38	gp38		
membrane protein		gp33				
membrane protein		gp34				
membrane protein		gp35				
dipeptidyl aminopeptidase/acylaminoacyl-peptidase		gp37				
recombinase/Integrase		gp40				
XRE family transcriptional regulator	gp30	gp41	gp39	gp39	gp38	gp32
XRE family transcriptional regulator		gp42	gp40	gp40	gp42	gp33
Cro/C1 family transcriptional regulator	gp31				gp43	
antirepressor AntA				gp41		
excisionase	gp32			gp42		
restriction endonuclease			gp42			
Rha family regulatory protein						gp35
antirepressor Rha	gp34		gp44		gp49	
antirepressor	gp38	gp49				gp38
XRE family transcriptional regulator						gp41
excisionase			gp45			
DNA repair protein RecN	gp42				gp52	
DNA recombinational protein RecT	gp44				gp54	
metallo-hydrolase	gp45				gp55	
primosome component protein	gp47				gp57	
DNA replication protein						gp47
replicative DNA helicase	gp48				gp58	gp48
DNA primase						gp49
DNA methylase			gp60			
single-stranded DNA binding protein, ERF superfamily		gp57				
single-stranded DNA binding protein		gp58				gp52
putative phosphomannomutase						gp54
transcriptional regulator AbrB			gp63	gp47		
replication terminator protein			gp64	gp48		
primosome, DnaD subunit		gp61				
DNA replication protein		gp62 ^4^				
chromosome segregation protein SMC			gp66 ^5^	gp50		
AAA domain ATPase			gp67 ^6^	gp51		
DEAD/DEAH box helicase			gp69	gp54		
ABC-type transport system			gp70			
DNA primase			gp71 ^7^	gp56		
DNA polymerase I with exonuclease domain						gp59
DNA polymerase family A						gp61
DEDDh 3′-5′ exonuclease						gp63
endodeoxyribonuclease		gp65	gp73	gp58		gp65
DNA-N-6-adenine methyltransferase					gp61	
DNA–cytosine methyltransferase		gp67			gp62	
DNA-cytosine methyltransferase					gp63	
resolvase RuvC	gp59				gp68	
ribonucleoside-diphosphate reductase subunit alpha						gp67
ribonucleoside-diphosphate reductase subunit beta						gp68
deoxyuridine 5′ triphosphate nucleotidohydrolase						gp69
antitoxin MazE						gp76
deoxynucleoside monophosphate kinase						gp80
RNA polymerase sigma factor			gp75	gp60		gp82
transcriptional regulator ArpU	gp61	gp73			gp70	
transcriptional activator RinA			gp77	gp61		
tRNA-Pro(tgg)				gp62		
serine recombinase					gp72	
membrane protein		gp74				
toxin HicA			gp79			
antitoxin HicB	gp62	gp75	gp80		gp74	
toxin HicA	gp63	gp76			gp75	
Putative transglycosylase	gp64	gp77	gp81			
HNH endonuclease/restriction endonuclease McrA				gp65		
HNH endonuclease	gp65	gp78	gp84	gp66		

^1^ Also has strong BLAST and CDD matches to bacteriocin; ^2^ Also has strong BLAST matches to transposase;.^3^ Also has strong BLAST matches to toxin-like protein, FAA hydrolase, RNA polymerase sigma factor, DNA processing protein DprA, inositol phosphorylceramide, DNA Smf single strand binding protein, transcriptional regulatory protein YclJ, toxin, and transposase; ^4^ Also has equally strong BLAST and CDD matches to chromosomal replication initiator protein DnaA; ^5^ Also has equally strong BLAST matches to DNA recombination protein RecF; ^6^ Also has strong BLAST matches to oxidoreductase, putative DNA helicase, putative RecA NTPase, ATP-dependent Lon protease; ^7^ Also has equally strong BLAST matches to RecA family ATPase.

**Table 4:** Pham distribution and composition of the portal protein, major capsid protein, and tape measure protein. Entries in boldface indicate clusters, with the number of phages in the cluster in parentheses.

	Pham 1	Pham 2	Pham 3	Pham 4
Portal	**Fern** (30) **Harrison** (2) Diane Hayley Vadim Vegas	Dragolir LincolnB Wanderer	Lily	**Halcyone** (8)
Major capsid	**Fern** (30) **Harrison** (2) Diane Hayley Vadim Vegas	Dragolir LincolnB Wanderer	Lily	**Halcyone** (8)
Tail tape measure	**Fern** (30)	**Harrison** (2) **Vegas** (6)	Lily	**Halcyone** (8)

**Table 5:** Best TBLASTN match of selected *P. larvae* phage major capsid proteins to *Paenibacillus larvae* phage major capsid proteins in bacterial chromosomes.

Phage	Best TBLASTN bacterial match	Accession #	E-value (AAI)
Fern	*Paenibacillus larvae* subsp. Larvae strain Eric I	CP019651	0.0(87%)
Harrison	*Paenibacillus larvae* subsp. Larvae strain Eric I	CP019651	0.0(100%)
Vegas	*Paenibacillus larvae* subsp. Larvae strain Eric I	CP019651	0.0(100%)
Dragolir	*Paenibacillus formosus strain NF2*	CP018145	0.0(72%)
Lily	*Paenibacillus larvae* subsp. Larvae strain Eric IV	CP019659	0.0 (99%)
Halcyone	*Paenibacillus larvae* subsp. Larvae strain Eric IV	CP019659	0.0 (100%)

## Supplementary Materials

The following are available online at http://www.mdpi.com/1999-4915/10/7/377/s1, Figure S1. Complete ANI distance matrix of all *P. larvae* phage genomes. Figure S2. Complete AAI distance matrix of all *P. larvae* phage large terminases. Figure S3. Complete AAI distance matrix of all *P. larvae* phage major capsid proteins. Figure S4. Complete AAI distance matrix of all *P. larvae* phage *N*-acetylmuramoyl-l-alanine amidases. Table S1. Complete list of all *P. larvae* phage protein phams and their members. Table S2. Complete list of all *P. larvae* phage genes and their function.
