# Supplementary figures and images for: Genomic Analysis of 48 Paenibacillus larvae Bacteriophages
Source: Viruses. 2018 Jul 19;10(7):377. doi: 10.3390/v10070377 (PMC6070908; doi:10.3390/v10070377)

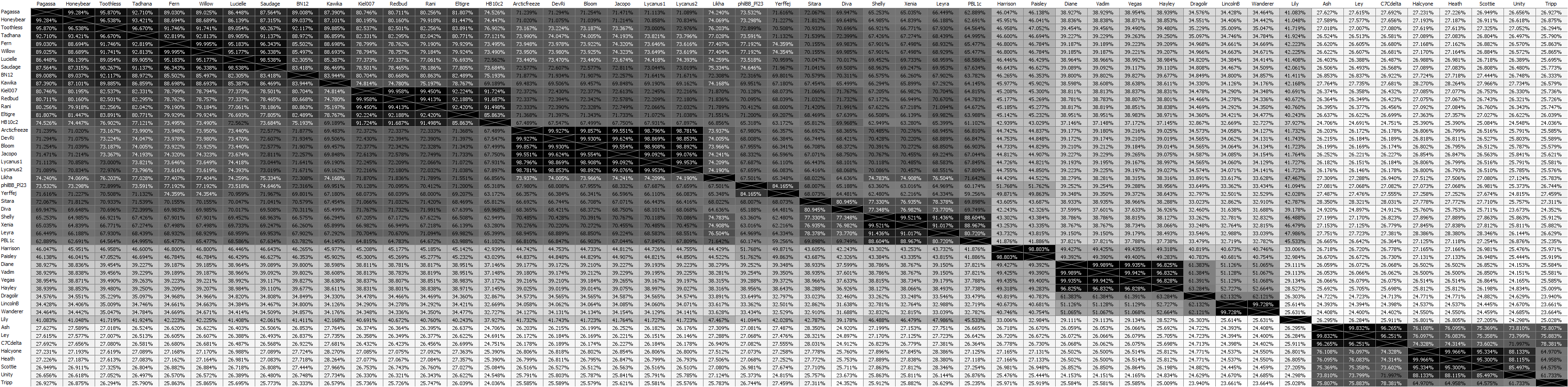

Supplement: Supplementary file 1 [file viruses-10-00377-s001.zip › viruses-328735-supplementary/Figure S1. Distance matrix final.png]

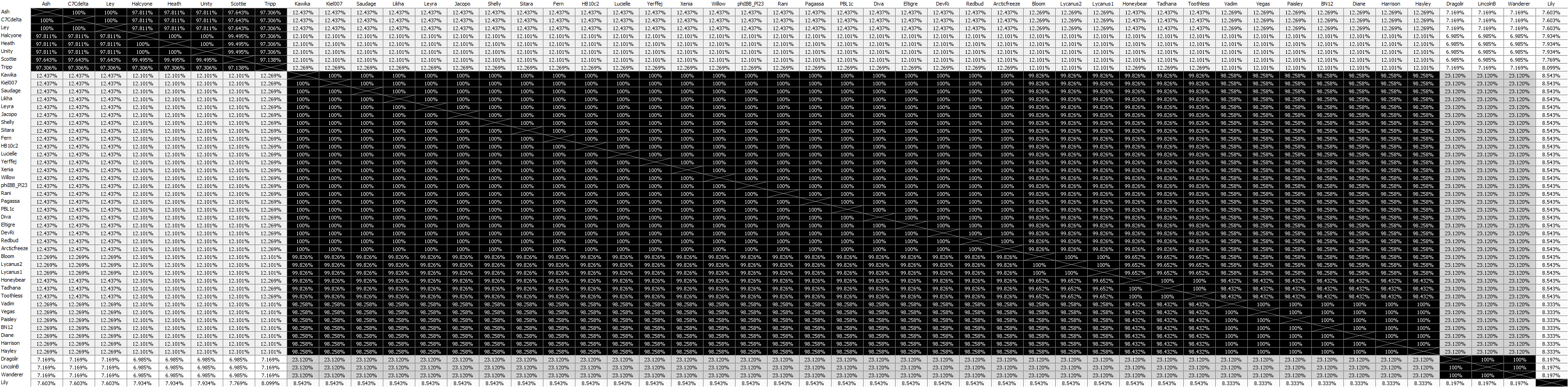

Supplement: Supplementary file 1 [file viruses-10-00377-s001.zip › viruses-328735-supplementary/Figure S2. Large terminase distance matrix full.png]

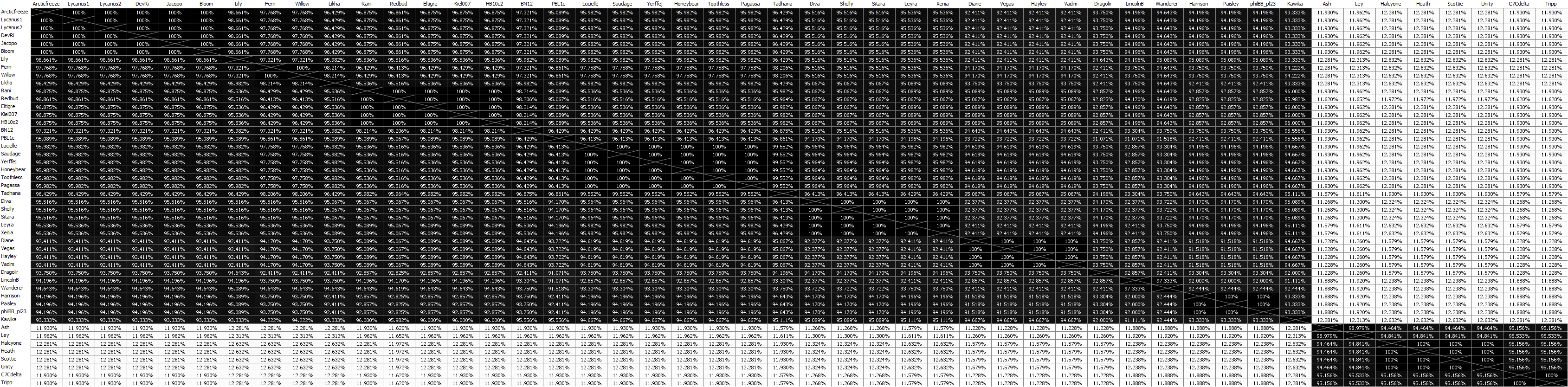

Supplement: Supplementary file 1 [file viruses-10-00377-s001.zip › viruses-328735-supplementary/Figure S3. Lysin distance matrix all.png]

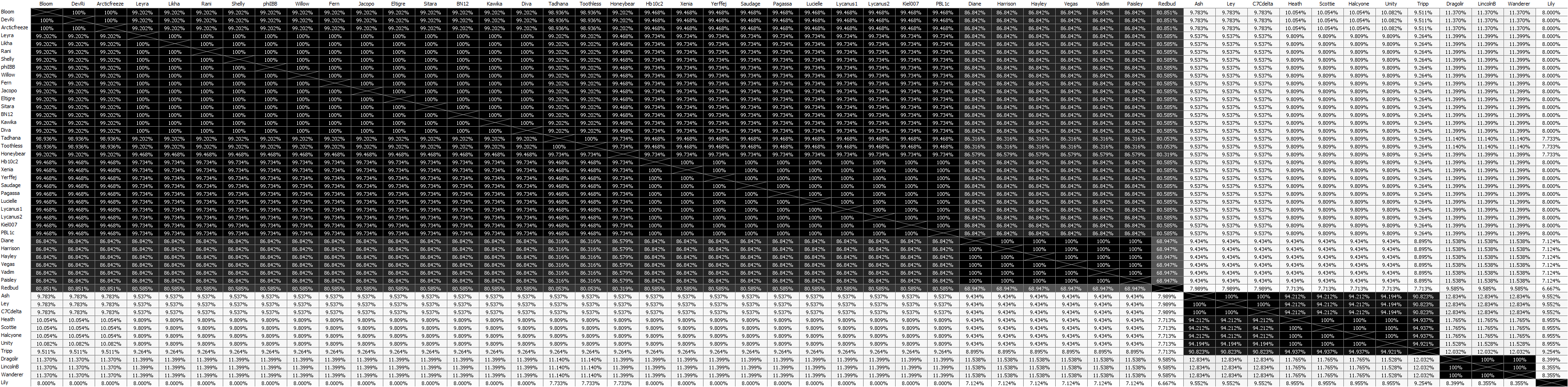

Supplement: Supplementary file 1 [file viruses-10-00377-s001.zip › viruses-328735-supplementary/Figure S4. MCP distance matrix all.png]
